# The Cultivation of Halophilic Microalgae Shapes the Structure of Their Prokaryotic Assemblages

**DOI:** 10.3390/microorganisms12101947

**Published:** 2024-09-26

**Authors:** Elena A. Selivanova, Michail M. Yakimov, Vladimir Y. Kataev, Yuri A. Khlopko, Alexander S. Balkin, Andrey O. Plotnikov

**Affiliations:** 1 Institute for Cellular and Intracellular Symbiosis of the Ural Branch of Russian Academy of Sciences, Orenburg Federal Research Center of the Ural Branch of Russian Academy of Sciences, 460000 Orenburg, Russia; vladimir0334@yandex.ru (V.Y.K.); 140374@mail.ru (Y.A.K.); balkinas@yandex.ru (A.S.B.); 2 Extreme Microbiology, Biotechnology and Astrobiology Group, Institute of Polar Research, The Institute of Polar Sciences of the National Research Council (ISP-CNR), 98122 Messina, Italy; mikhail.iakimov@cnr.it

**Keywords:** halophilic microalgae, prokaryotic assemblages, hypersaline habitats, 16S rRNA amplicon high-throughput sequencing

## Abstract

The influence of microalgae on the formation of associated prokaryotic assemblages in halophilic microbial communities is currently underestimated. The aim of this study was to characterize shifts in prokaryotic assemblages of halophilic microalgae upon their transition to laboratory cultivation. Monoalgal cultures belonging to the classes Chlorodendrophyceae, Bacillariophyceae, Trebouxiophyceae, and Chlorophyceae were isolated from habitats with intermediate salinity, about 100 g/L, nearby Elton Lake (Russia). Significant changes were revealed in the structure of algae-associated prokaryotic assemblages, indicating that microalgae supported sufficiently diverse and even communities of prokaryotes. Despite some similarities in their prokaryotic assemblages, taxon-specific complexes of dominant genera were identified for each microalga species. These complexes were most different among *Alphaproteobacteria*, likely due to their close association with microalgae. Other taxon-specific bacteria included members of phylum *Verrucomicrobiota* (*Coraliomargarita* in assemblages of *Navicula* sp.) and class *Gammaproteobacteria (Salinispirillum* in microbiomes of *A. gracilis*). After numerous washings of algal cells, only alphaproteobacteria *Marivibrio* remained in all assemblages of *T. indica*, likely due to a firm attachment to the microalgae cells. Our results may be useful for further efforts to develop technologies applied for industrial cultivation of halophilic microalgae and for developing approaches to obtain new prokaryotes with a microalgae-associated lifestyle.

## 1. Introduction

Large-scale microalgae cultivation is one of the current priorities in aquaculture, aiming not only to combat the increasing greenhouse gas production by capturing the immense amount of carbon dioxide, but also to produce a biomass with considerable nutritional value [1,2]. In addition to easily digestible proteins, it contains many valuable nutrients such as vitamins, polyunsaturated fatty acids, carotenoids, etc. [3,4]. Considering that axenic microalgae cultures are too unrealistic, unsuitable, and labor-intensive for use, always exposed to the risk of obvious contamination in large-scale and long-term applications, the isolation of algological pure cultures of microalgae with their subsequent application in biotechnology is the first step towards obtaining a valuable and stable biotechnological product [5]. In precise terms, algologically pure (monoalgal) culture means mechanical (under microscopy examination) selection of a single microalga cell floating in at least 2–5 µL of environ, obviously containing a great number of prokaryotic cells. It has been suggested that the microenvironment of prokaryotes associated with algae provides protection to the latter from many environmental disturbances [6].

In 1972, Bell and Mitchell proposed the term “phycosphere” to describe an area surrounding the microalgal cell and extending for some distance on the µm scale, within which bacterial growth is stimulated by algal extracellular products [7]. Recent studies have shown that more complex interactions occur between marine microalgae and their associated bacteria [8,9,10,11]. In addition to interactions based on the uptake by prokaryotes of dissolved organic matter released by microalgae, a diverse set of complex mutual chemical signaling and metabolite exchanges were detected [8,9,10,11]. Historically, associations of microalgae with prokaryotes have been studied intensively in freshwater and marine ecosystems, as well as in some extreme habitats [12,13,14]. Long-term cultures of microalgae that maintain symbiotic relationships with bacteria are also the focus of research and therefore used as a convenient model for studying trophic interactions in complex microbial communities, as well as the stimulation of algae growth and mutual suppression and competition for nutrients [15,16,17,18,19]. Interactions based on an uptake by prokaryotes of microalga-derived dissolved organic matter, exchange of vitamins, iron compounds, action of auxins, antimicrobial compounds, etc., have important ecological consequences for water communities. In general, the relations can be divided into competitive, synergistic, and parasitic [20]. Bacteria can either compete with microalgae for limited resources or even produce toxic substances against microalgae. Moreover, prokaryotes can either enhance or suppress the growth and development of microalgae, influencing the process known as “algal blooming” [18,19,21]. Certainly, the character of microbial interactions should be considered in large-scale microalgae cultivation due to possible impacts on the culture stability, biomass growth, and yield of useful products. Knowledge on the composition of the microalga microbiomes and understanding their formation modes will help to support the sustainable stable growth of microalgae for biotechnological application and industrial use [6]. As mentioned above, the biotechnological role of microalgae is growing, particularly as components of food supplements, animal feed, and medicines, as well as for energy production and phytoremediation [3,4,15]. 

Halophilic microalgae, despite the technological difficulties of their use, are promising objects for biotechnology due to their ability to accumulate a significant amount of carotenoids, compatible solutes, lipids, and polyunsaturated fatty acids compared to freshwater and marine algae [22,23,24,25]. However, associations of microalgae with prokaryotes in hypersaline conditions have been studied to a much lesser extent. This is especially true for the intermediate salinity level, at which the microbiomes of halophilic microalgae have been practically unstudied. In reservoirs with a salinity of about 100 g/L, the composition of microalgae differs from both seawater and reservoirs with a salinity more than 200 g/L. In contrast to conditions of extreme salinity where the diversity of microalgae is sharply reduced and limited to extreme halophiles, under the intermediate salinity level, more diverse communities of chlorophyte algae and diatoms develop, many of which are poorly studied or even unidentified [26,27].

Taking into account all mentioned above, in this study, we obtained a couple of samples from inland saline waters, isolated monoalgal cultures from them, and analyzed the algae microbiomes compared to natural prokaryotic communities using DNA metabarcoding. We attempted to address the following main questions in this study:

- How do the diversity and composition of the prokaryotic communities change upon isolation of halophilic algae in laboratory cultures?
- Does the taxonomic affiliation of halophilic microalgae determine the composition of their prokaryotic assemblages?
- Are the prokaryotic assemblages of halophilic microalgae composed randomly, or are there specific prokaryotic taxa featuring microalgal species?

## 2. Materials and Methods

### 2.1. Sampling, Alga Cultures, and Growth Conditions

Samples were collected in 2019 from two hypersaline sites in the basin of Lake Elton, Volgograd region, Russia, the mouth of the Malaya Smorogda River, also named Malaya Samoroda River (MS), with salinity of 110 ppt (49.0960N 46.7333E), and an ephemeral pond near the Solyanka River (EPS) with salinity 100 ppt (49.1845N 46.5946E). Water samples of 0.5 L were collected at each site from a depth of 10 cm in sterile bottles. Sampled water of 50–100 mL volume was filtered sequentially through 5.0 and 0.22 µm membranes immediately after collection. The membranes were placed in sterile Eppendorf tubes with 200 μL of DNA/RNA Shield™ (Zymo Research, Irvine, CA, USA), transported to the laboratory, and stored at −80 °C until DNA extraction. Remaining water from the samples was transported to the laboratory, examined under an Axioskop light microscope (Carl Zeiss, Oberkochen, Germany), and used for microalgae isolation and the preparation of the cultivation medium. A total of 26 monoalgal cultures assigned to 5 species (4–5 cultures of each species from each site) were obtained by direct isolation of single cells under a Nikon TS2 inverted microscope (Nikon, Tokyo, Japan) with glass Pasteur pipettes (Table 1). Isolation of monoalgal cultures of *T. indica* from the MS sample was complicated by a huge amount of tiny *Picochlorum* sp. cells. Thus, we had to ‘wash’ the *T. indica* cells through several (4–5) sequential transfers from one drop of sterile medium to another under microscope examination. At the initial stages, clonal cultures were grown in bacteria-free natural water obtained by filtration through the 0.22 μm membrane with subsequent autoclaving. Then, the algae were cultivated at 25 °C under periodic 5 klx illumination with luminescent lamps (12 h—day, 12 h—night) in modified OPS medium that contained 82.3 g NaCl, 17.0 g MgSO_4_, 2.5 g KNO_3_, 0.2 g K_2_HPO_4_, and 1.0 g NaHCO_3_ per 1 L (salinity 100 g/L). The purity of the obtained cultures was controlled microscopically and using 18S metabarcoding. The cultivation lasted three months; during that time, the cultures underwent five transfers to fresh medium. As a control, the samples without microalgae cells were picked up with Pasteur pipettes under a Nikon TS2 inverted microscope (Nikon, Tokyo, Japan) to assess a prokaryotic community in the absence of algae. The mineral medium applied for microalgae cultivation was used for the control samples to evaluate the specific impact of algae on the structure of prokaryotic communities, and to identify possible contamination of the algal cultures in the laboratory. 

### 2.2. DNA Extraction

Total DNA was extracted from the microalgae cultures at the late logarithmic growth phase, as well from the control samples and natural samples (eDNA). Cultures and control samples (1.5 mL) were centrifuged for 5 min at 14,000 rpm and 6 °C. Supernatants were removed up to 100 μL residual volume. The pellets were homogenized on a TissueLyser LT (Qiagen, Hilden, Germany) using the Lysing Matrix Y (MP Biomedicals, Solon, OH, USA) for 1 min at 50 Hz and supplemented with 50 μL of TE-buffer. After centrifugation for 5 min at 14,000 rpm, the supernatants were transferred to clean tubes and heated at 95 °C for 10 min. The samples were supplemented with TE-buffer up to a volume of 800 μL. Total DNA from the extracts was cleaned and concentrated using a NucleoSpin^®^ gDNA Clean-up XS column kit (Macherey-Nagel GmbH & Co. KG, Düren, Germany). For eDNA extraction, each membrane was transferred into a Lysing Matrix E tube (MP Biomedicals, LLC, Solon, OH, USA) with the addition of 2× volume of tris-saline buffer (1 M Tris-HCl; 0.5 M EDTA; 5 M NaCl; MQ) and homogenized for 5 min at 50 Hz (TissueLyser LT, Qiagen, Hilden, Germany). Then, biomass on membranes was enzymatically digested (lysozyme, proteinase K, SDS in total conc. 1%) and DNA was extracted by phenol/chloroform (phenol/chloroform 1:1 *v*/*v*; chloroform/isoamyl alcohol 24:1 *v*/*v*) and precipitated from the aqueous phase by a threefold volume of absolute ethanol by adding 10% *v*/*v* 10 M ammonium acetate at −20 °C overnight. After centrifugation and double washing with 80% ethanol, DNA was air-dried and dissolved in 30 μL of autoclaved deionized water. To estimate possible contamination, negative controls were used, for which autoclaved deionized water (100 μL) was treated along with experimental samples as described above. The concentration, quality, and quantity of DNA were estimated by electrophoresis in 1% agarose gel and via photometry using a NanoDrop 8000 (Thermo Fisher Scientific Inc., Waltham, MA, USA). 

### 2.3. Preparation of DNA Libraries and Sequencing

16S rDNA libraries for high-throughput sequencing were prepared according to the Illumina protocol (Part no. 15044223, Rev. B). DNA amplification was performed using S-D-Bact-0341-b-S-17 (forward) and S-D-Bact-0785-a-A-21 (reverse) primers targeting the V3 and V4 regions of the 16S rRNA gene [28]. 18S rDNA libraries for algae identification were prepared from all 26 monoalgal cultures using forward TAReuk454FWD1 and reverse TAReukRev3 primers targeting the hypervariable V4 region of the 18S rRNA gene [29], producing amplicons with lengths of about 500 bp. The libraries were sequenced on the MiSeq platform (Illumina, San Diego, CA, USA) using a 2 × 300 bp paired-end v3 MiSeq Reagent Kit in the “Persistence of microorganisms” Science Resource Centre, the Institute for Cellular and Intracellular Symbiosis, the Ural Branch of the Russian Academy of Sciences. 

### 2.4. Bioinformatic Pipeline 

Paired-end reads were merged with a minimal overlap of 30 bp, a *p*-value of 0.0001, and a Q-score of 30 using PEAR v. 0.9.10 [30]. At the next stage, if there were adapters, they were trimmed by the program Trimmomatic V. 0.36 [31]. Subsequent treatment of merged and trimmed reads was conducted using the UPARSE algorithm of the USEARCH v. 10.0.240 program [32] and included quality filtering and amplicon size selection (400 bp—minimal size for 16S rRNA data; 360 bp—minimal size for 18S rRNA data). The reads with Ns and a maximum number of expected errors above one were discarded during the filtering procedure. The filtering quality was evaluated with FastQC v. 0.11.7. As a result of dereplication and clustering with the UPARSE algorithm from USEARCH, operational taxonomic units (OTUs) were formed, while singletons and doubletons were removed. For clustering of OTUs, we used a 97% threshold. Chimeric sequences were detected and removed using USEARCH v. 10.0.240 at the stage of OTU clustering [33]. The resulting OTUs were globally aligned to merged reads that resulted in an OTU table. Every sample was checked for contaminant 16S rRNA gene fragments that originated from bacterial cells or DNA, which were possibly present in reagents, water, air, laboratory plastic, or other contaminants on hands. OTUs resulting from contaminant 16S rRNA fragments were identified at a similarity level of 98% and removed via the USEARCH command ublast by matching the sequences from the samples with the positive control and negative control ones. The OTUs corresponding to chloroplast DNA were also excluded from the library for subsequent analysis. Classification of the OTUs was conducted against the RDP database with an 80% threshold [34]. OTUs with low support were additionally checked using the NCBI GenBank database (https://www.ncbi.nlm.nih.gov/genbank/, accessed on 1 May 2024) and built-in tool BLAST (https://blast.ncbi.nlm.nih.gov/Blast.cgi, accessed on 1 May 2024). Taxonomic affiliation is indicated in accordance with the NCBI Taxonomy Database (https://www.ncbi.nlm.nih.gov/taxonomy/, accessed on 1 May 2024).

### 2.5. Statistical Analysis and Visualization

After bioinformatic processing, the data normalized by total sum scaling were analyzed using the MicrobiomeAnalyst tool (https://www.microbiomeanalyst.ca/, accessed on 1 May 2024) [35]. The observed number of OTUs and Chao1, Shannon, and Simpson indices were calculated using MicrobiomeAnalyst [35]. Principal coordinate analysis (PCoA) 2D plots demonstrating the ordination of the samples were constructed using permutational multivariate analysis of variance (PERMANOVA) based on the Bray–Curtis dissimilarities using MicrobiomeAnalyst [35]. Mantel’s test was carried out, applying the abundance tables of dominant prokaryote genera depending on the presence of different microalgae species, and visualized using the ‘LinkET’ R package v. 0.0.7.4 in the R 4.4.0 environment [36]. Bar charts were created using Microsoft Excel 2019 v. 1808. Bubble charts were built using the package ggplot2 [37]. 

## 3. Results

### 3.1. Characteristics of the Isolated Monoalgal Cultures 

First, we examined under an Axioskop light microscope (Carl Zeiss, Oberkochen, Germany) several microalgal communities sampled from two hypersaline habitats, and obtained a number of monoalgal cultures. Green microalgae were abundant in the sample from the ephemeral pond near the Solyanka River (EPS) with a salinity of 100 ppt. Direct isolation of individual algal cells resulted in clones of three different morphotypes. According to the sequences of the V4 region of their 18S rRNA genes, the obtained cultures were identified as *Dunaliella* sp., *Tetraselmis indica*, and *Asteromonas gracilis* (Table 1). As for the sample from the Malaya Smorogda River (MS) with 110 ppt salinity, there were diatoms in mass together with green microalgae. Clone cultures of a diatom *Navicula* spp., green alga *Picochlorum* sp. and *Tetraselmis indica* were obtained and identified in the same way. Results of 18S rRNA gene sequencing clearly indicated that the cultures of each species could be assigned to a single common OTU, including both MS and EPS cultures of *T. indica* (Table 1). However, MS cultures of *Navicula* sp. were assigned to two barely distinguishable OTUs, which had similarity of 99.28 and 98.89 with the closest homologue from NCBI, *Navicula salinicola* MT012298.

### 3.2. General Characteristics of the 16S rDNA Metabarcoding Data

The composition of the natural prokaryotic communities, assemblages associated with monoalgal cultures after five passages through OPS laboratory medium, and control ones was analyzed using DNA metabarcoding. The number of paired-end reads with a maximum length of 2 × 300 base pairs (bp) varied from 10,432 to 80,277 per sample (Tables S3 and S4). The number of high-quality reads obtained after dereplicating, filtering, and removal of chimeric sequences varied from 4020 to 36,431 per sample. The total amount of prokaryotic OTUs ranged from 2 to 196 per sample after clustering at the 97% level and the removal of singletons and doubletons, contaminant sequences, and OTUs assigned to chloroplasts (Tables S3 and S4). 

### 3.3. Alpha-Diversity and Taxonomic Composition of the Natural Prokaryotic Communities from the Hypersaline Sites

**Figure 1:**
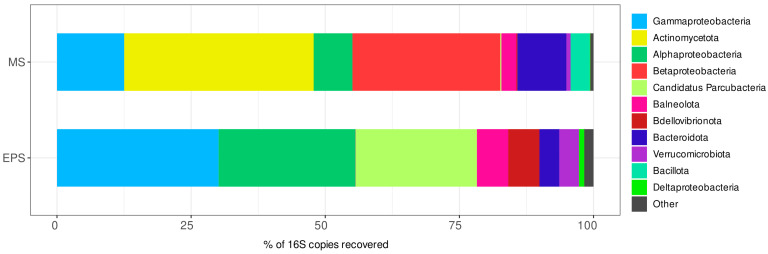
Taxonomic composition of water prokaryotic communities from the hypersaline sites the ephemeral pond near the Solyanka River (EPS) and the Malaya Smorogda River (MS) at the phylum level. Classes are indicated for phylum *Pseudomonadota* only. The top 10 most abundant taxa in every community are shown.

The structure of natural prokaryotic communities in the EPS and MS water samples was quite different despite their similar salinity. A total of 196 OTUs belonging to 97 genera were identified in the EPS community. The Shannon and Simpson biodiversity indices for the EPS prokaryotic community were 2.96 and 0.87, respectively. Classes *Gammaproteobacteria* and *Alphaproteobacteria* and a phylum of uncultivated bacteria, “*Candidatus* Parcubacteria”, were predominant (Figure 1). Interestingly, sequences belonging to unclassified *Francisellaceae* dominated among *Gammaproteobacteria* (Table S1). Genus *Roseivirga* dominated among *Alphaproteobacteria* together with unclassified OTUs. In addition, phyla *Bacteroidota*, *Balneolota*, *Verrucomicrobiota*, and *Bdellovibrionota*, represented mainly by genus *Halobacteriovorax,* had significant proportions. *Verrucomicrobiota* contained the dominant genera *Puniceicoccus* and *Coraliomargarita*. Phyla *Balneolota* and *Bacteroidota* were represented by the dominant genera *Gracilimonas* and *Roseivirga*, respectively.

In the MS samples, 117–134 OTUs were detected, which belonged to 70–75 genera. The Shannon and Simpson biodiversity indices were 2.70–2.77 and 0.84–0.86, respectively. More than a third of the prokaryotic reads were attributed to phylum *Actinomycetota*; classes *Betaproteobacteria* and *Gammaproteobacteria* also made up a large proportion (Figure 1). Phyla *Balneolota*, *Bacillota*, and *Bacteroidota* occupied slightly less proportions. The dominant actinomycetes were represented by genera *Rhodoluna*, *Pontimonas*, and *Longivirga* (Table S2). *Betaproteobacteria* were represented only by genus *Bordetella*. The most abundant genera of *Gammaproteobacteria* were *Spiribacter* and *Wenzhouxiangella* (*Chromatiales*). Among *Bacteroidota*, the dominant genera included *Psychroflexus*, *Mesohalobacter*, and *Owenweeksia*. Genera *Roseovarius* (*Alphaproteobacteria*), *Rhodohalobacter* (*Balneolota*), and unclassified *Erysipelotrichaceae* (*Bacillota*) also dominated in the MS community.

### 3.4. Alpha-Diversity and Taxonomic Composition of the Control Prokaryotic Communities

Most control samples of prokaryotic communities grown in mineral medium without algae did not produce amplicons with prokaryotic primers. This indicates a very insignificant growth of prokaryotes in mineral medium without microalgae. Ultimately, we managed to obtain amplicons from only two control EPS samples of ten, and for one control MS sample of six.

**Figure 2:**
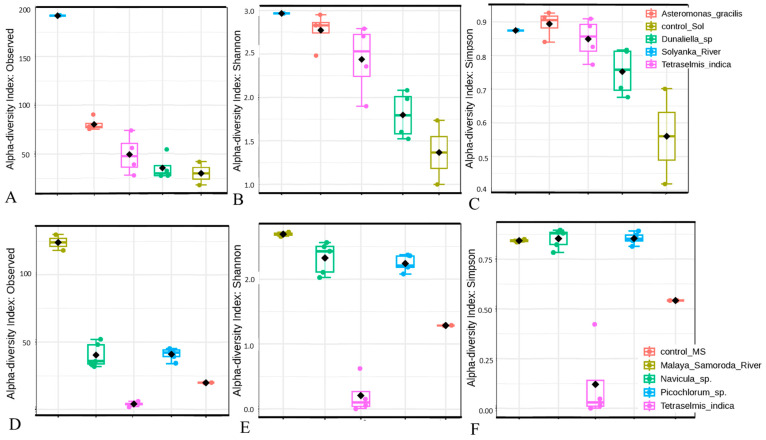
Alpha-diversity metrics for prokaryotic assemblages associated with the monoalgal cultures derived from the ephemeral pond near the Solyanka River (**A**–**C**), and from the Malaya Smorogda River (**D**–**F**) in comparison with natural prokaryotic communities (Solyanka_River, Malaya_Samoroda_River) and control samples (control_Sol, control_MS). Black diamonds mean average values.

The taxonomic richness and alpha-diversity of both EPS and MS prokaryotic communities decreased sharply upon the start of laboratory cultivation without microalgae. Thus, in the EPS control samples, a significant decrease in taxonomic richness was observed; namely, 21–37 OTUs belonged to 12–19 genera and 5–6 classes of prokaryotes (Figure 2A, Table S3). The Shannon and Simpson indices were 1.00–1.74 and 0.42–0.70, respectively (Figure 2B,C). The same trend was noted for the MS control sample, where 23 OTUs belonging to 11 genera and 4 classes of prokaryotes were observed (Figure 2D, Table S4). The Shannon and Simpson indices were 1.28 and 0.54, respectively (Figure 2E,F). 

While major phyla observed in the natural communities disappeared in the EPS and MS control samples, *Pseudomonadota*, occupying a lower proportion in the natural communities, increased its abundance. This accompanied opposite changes in percentages of the main *Pseudomonadota* classes, namely a sharp increase in *Gammaproteobacteria* and a decrease in *Alphaproteobacteria*. The taxonomic composition of all control samples was characterized by the considerable predominance of genus *Marinobacter*, more than 50%. For both control samples, *Alphaproteobacteria* were presented by the same dominant genera as in natural communities, for instance, *Roseovarius*. An increase in the proportion of class *Saprospiria* was also noted for most control samples. The other taxa were detected occasionally (Tables S1 and S2). 

### 3.5. Alpha-Diversity of Prokaryotic Assemblages Associated with the Monoalgal Cultures

The taxonomic richness of prokaryotic assemblages in the monoalgal cultures decreased upon transition to laboratory cultivation. In the EPS monoalgal cultures, we found only 30–77 OTUs assigned to 14–45 genera and 3–10 classes (Figure 2A, Table S3). At the same time, the Shannon and Simpson indices decreased to a lesser extent and remained at relatively high levels compared to the control communities. The highest diversity indices of prokaryotic assemblages were recorded in the EPS cultures of *A. gracilis* (Shannon index 2.48–2.95, Simpson index 0.84–0.93) (Figure 2B,C). The lowest diversity indices were found in the assemblages of *Dunaliella* sp. (Shannon index 1.52–2.08, Simpson index 0.68–0.82). 

Prokaryotic assemblages of the MS alga cultures showed similar trends in taxonomic richness and alpha-diversity as described above. In particular, 2–46 OTUs from 2–8 classes were found in the MS alga cultures (Figure 2D, Table S4). The Shannon index for bacterial assemblages of both *Navicula* sp. (2.03–2.56) and *Picochlorum* sp. (2.08–2.37) cultures was slightly different from the MS natural communities (2.70–2.77) in contrast to its very low value in the control community (1.28) (Figure 2E,F). The same trend was noted for the Simpson index, which was similar between prokaryotic assemblages of both *Navicula* sp. (0.78–0.90) and *Picochlorum* sp. (0.81–0.87) cultures. Prokaryotic assemblages associated with *T. indica* differed sharply from those in the other algal cultures by low diversity indices due to the presence of only 2–5 OTUs per culture. This phenomenon was probably determined by a thorough washing of single algal cells through repeated (4–5 times) transfers in a sterile medium to remove the *T. indica* cultures of small cells of predominant *Picochlorum* spp.

### 3.6. Beta-Diversity of Prokaryotic Assemblages Associated with the Monoalgal Cultures

**Figure 3:**
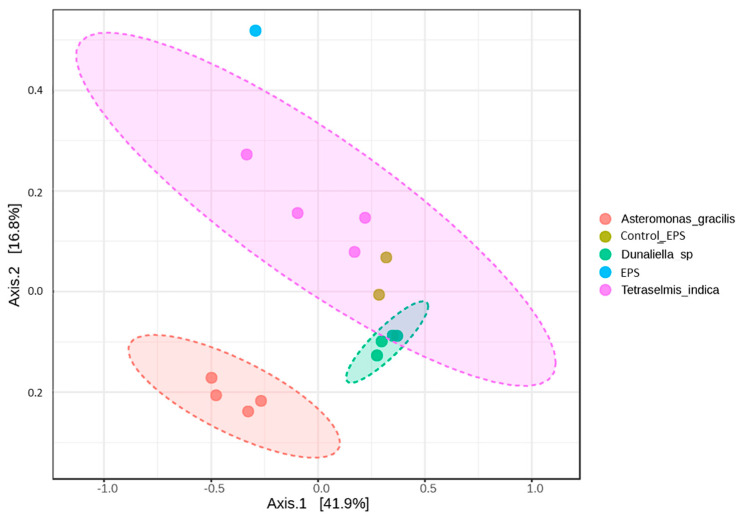
PCoA 2D plot based on the Bray–Curtis metrics of the prokaryotic assemblages associated with the monoalgal cultures derived from the ephemeral pond near Solyanka River, natural prokaryotic community (EPS), and control samples (Control_EPS); PERMANOVA F-value: 5.6194; *p*-value: 0.001.

PCoA analysis based on the Bray–Curtis metrics of the prokaryotic assemblages associated with the EPS monoalgal cultures revealed three separated clusters belonging to *Asteromonas gracilis*, *Tetraselmis indica*, and *Dunaliella* sp., respectively (Figure 3). The control assemblages were closer to those of *Dunaliella* sp. The dendrogram built on the Bray–Curtis metrics demonstrated the same clusters (Figure S1). 

**Figure 4:**
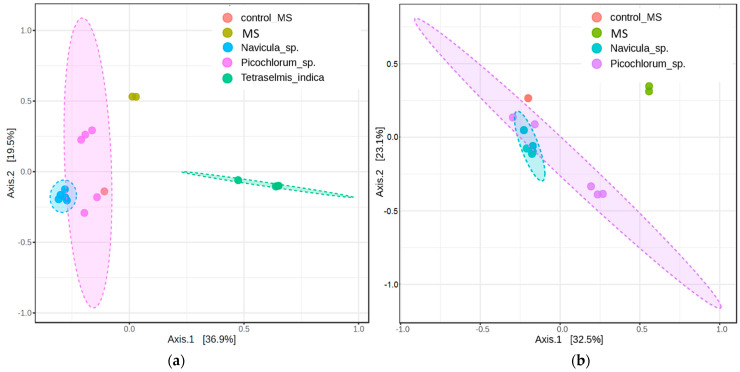
PCoA 2D plots based on the Bray–Curtis metrics of the prokaryotic assemblages associated with the monoalgal cultures derived from Malaya Smorogda River, natural prokaryotic communities (MS), and control sample (control_MS): (**a**) *Tetraselmis indica* assemblages are included; PERMANOVA F-value: 8.8769; *p*-value: 0.001: (**b**) *Tetraselmis indica* assemblages are not included; PERMANOVA F-value: 6.4412; *p*-value: 0.001.

Prokaryotic assemblages associated with the MS cultures of *Picochlorum* sp. and *Navicula* sp. were close to each other, whereas the assemblages of *T. indica* formed a separate cluster located far from them (Figure 4 and Figure S2). After removing the *T. indica* assemblages from the analysis, the differences between the natural prokaryotic community and the alga assemblages became more evident.

### 3.7. Taxonomic Composition of the Prokaryotic Assemblages Associated with the Monoalgal Cultures

#### 3.7.1. Taxonomic Composition of the Prokaryotic Assemblages Associated with the Monoalgal Cultures from the Ephemeral Pond near Solyanka River (EPS)

**Figure 5:**
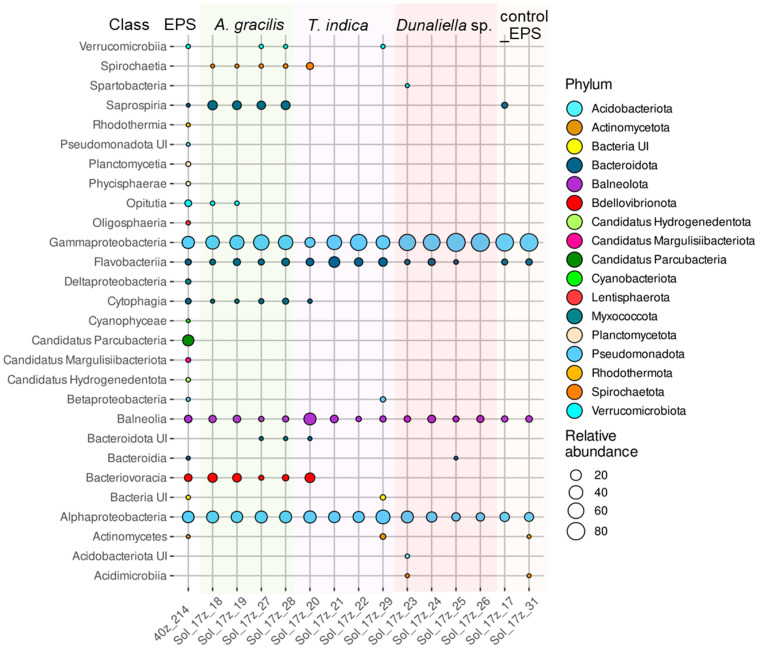
Relative abundances of prokaryotic classes belonging to different phyla in the assemblages associated with the monoalgal cultures derived from the ephemeral pond near Solyanka River.

Phyla *Cyanobacteriota*, “*Candidatus* Hydrogenedentota”, *Lentisphaerota*, “*Candidatus* Parcubacteria”, *Planctomycetota*, and *Rhodothermota* and class *Deltaproteobacteria* disappeared from the assemblages of the EPS monoalgal cultures upon laboratory cultivation compared to the natural EPS community (Figure 5). These taxa constituted a small part of the natural community, except for “*Candidatus* Parcubacteria” with a great relative abundance 22.6% (Figure 5, Table S1).

Phylum *Bacteroidota* dominated and its proportion increased significantly in assemblages with *A. gracilis* and *T. indica* (Figure 5, Table S1). However, class *Bacteroidia* assigned to this phylum disappeared, and class *Cytophagia,* represented by a single genus, *Marivirga,* was maintained only in the cultures of *A. gracilis*. In contrast, class *Flavobacteriia* retained its relative abundance in most monoalgal cultures, and increased its share in assemblages of *T. indica*. In most monoalgal cultures, one or two genera of flavobacteria were present as dominants including *Brumimicrobium*, *Salibacter*, *Mesohalobacter*, *Muricauda,* and *Psychroflexus* (Table S1). Their distribution was largely occasional; only *Mesohalobacter* was detected in all assemblages of *A. gracilis*. In addition, class *Saprospiria,* represented by a single OTU of unclassified *Saprospirales*, exclusively increased its percentage in association with *A. gracilis*. 

Phylum *Balneolota* maintained its share in the EPS monoalgal cultures, and was represented by two genera, *Gracilimonas* and *Rhodohalobacter* (Table S1).

The proportion of *Pseudomonadota* increased in the EPS monoalgal cultures (Figure 5, Table S1). It was represented mainly by classes *Alphaproteobacteria* and *Gammaproteobacteria*. *Alphaproteobacteria* had a similar high proportion in the assemblages of *A. gracilis* and *T. indica* as in the natural community, while its share decreased in association with *Dunaliella* sp. Taxonomic composition of *Alphaproteobacteria* differed in the natural community and monoalgal cultures (Table S1). Along with *Roseovarius* and unclassified *Rhodobacteraceae,* which dominated in the natural community, *Rhodovulum*, *Roseivivax*, *Marivibrio*, and *Thalassospira* dominated in the assemblages of *A. gracilis*. In all assemblages of *T. indica,* genera *Roseovarius* and *Thalassospira* dominated. In assemblages of *Dunaliella* sp. genus *Rhodovulum* was characterized by the highest relative abundance.

The relative abundance of class *Gammaproteobacteria* increased, especially in the cultures of *Dunaliella* sp. (Table S1). Genus *Marinobacter,* presented by one OTU closely related to *Marinobacter adhaerens* (MN595037), dominated in all samples, although its proportion in the natural community was insignificant (Table S1). The dominant genus *Spiribacter* reached the highest relative abundance in assemblages of *Dunaliella* sp. Another representative of order *Chromatiales*, genus *Wenzhouxiangella*, was detected only in association with *A. gracilis*, although its relative abundance was significantly lower compared to *Spiribacter*. Only assemblages of *A. gracilis* were featured with dominant genera *Salinispirillum* and *Saccharospirillum*. The hydrocarbon-degrading genus *Alloalcanivorax* was also detected in association with *A. gracilis* and *T. indica*. Methylotrophic Gammaproteobacteria *Methylophaga* were present in assemblages of all monoalgal cultures, and their relative abundance was greater than in the natural community, reaching 20.3% in association with *T. indica.*

In some monoalgal cultures, the predatory bacterium *Halobacteriovorax*, a genus of phylum *Bdellovibrionota*, persisted and its proportion even increased. In those assemblages, the proportion of *Gammaproteobacteria* decreased, perhaps due to predation.

#### 3.7.2. Taxonomic Composition of the Prokaryotic Assemblages Associated with the Monoalgal Cultures from Malaya Smorogda River (MS)

**Figure 6:**
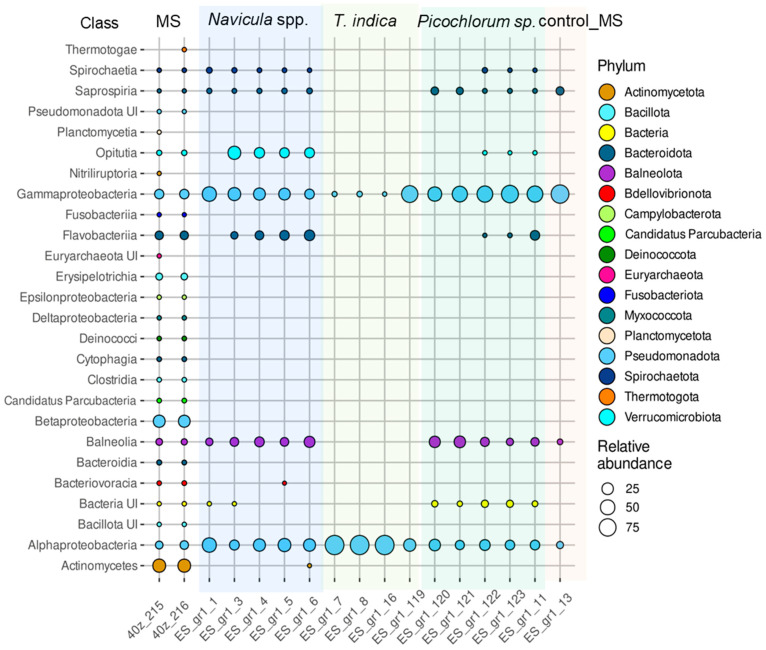
Relative abundances of prokaryotic classes belonging to different phyla in the assemblages associated with the monoalgal cultures derived from Malaya Smorogda River.

Compared to the natural MS community, many phyla completely disappeared in the MS monoalgal cultures including phyla *Euryarchaeota*, *Actinomycetota* (whose dominance in the natural community was 35%), *Campilobacterota*, *Deinococcota*, *Bacillota*, *Fusobacteriota*, “*Candidatus* Parcubacteria”, *Planctomycetota*, and *Thermotogota*, class *Deltaproteobacteria* and unclassified *Pseudomonadota* (Figure 6, Table S2). Although *Bacteroidota* retained its abundance in the algal assemblages, classes *Cytophagia* and *Bacteroidia* disappeared. At the same time, the proportion of phyla *Balneolota* and *Pseudomonadota* increased in associations with microalgae. An increase in the proportion of *Verrucomicrobiota* was noted only in assemblages of diatoms.

The dominance of phylum *Bacteroidota* in most monoalgal cultures was accompanied with the prevalence of classes *Flavobacteriia* and *Saprospiria* (Figure 6, Table S2). The relative abundance of class *Flavobacteriia* reached 13 and 18% in the *Picochlorum* sp. and *Navicula* sp. cultures, respectively. However, the genus composition of *Flavobacteriia* in assemblages of microalgae was different (Table S2). For example, *Psychroflexus* was the dominant genus in few assemblages, whereas *Salibacter* or *Owenweeksia* dominated in others. Class *Saprospiria* was found in all *Navicula* sp. and *Picochlorum* sp. cultures. Similar to the EPS monoalgal assemblages, the proportion of *Balneolota*, represented by *Gracilimonas* and *Rhodohalobacter*, increased in association with *Navicula* sp. and *Picochlorum* sp. Genus *Roseovarius* was a common dominant Alphaproteobacteria for assemblages of both *Navicula* sp. and *Picochlorum* sp. Genus *Saliniramus* and unclassified *Rhodovibrionaceae* were observed in all *Navicula* sp. cultures. Genera *Marivibrio*, *Tepidicaulis*, and unclassified *Rhodovibrionaceae* dominated in some *Picochlorum* sp. assemblages, whereas *Saliniramus* and *Oceanicaulis* prevailed in others. The relative abundance of *Marinobacter,* presented by the same OTU as in the EPS assemblages, was significantly larger in association with *Navicula* sp. and *Picochlorum* sp. Also, genus *Spiribacter* accounted for a significant proportion in association with *Navicula* sp. and *Picochlorum* sp. Genus *Methylophaga* dominated in all *Picochlorum* sp. cultures and reached 39.2%.

Genus *Wenzhouxiangella* was present only in association with *Navicula* sp. The hydrocarbon-degrading genus *Alloalcanivorax* was present in some assemblages of *Navicula* sp. and *Picochlorum* sp., although its relative abundance was rather low. Genus *Coraliomargarita (phylum Verrucomicrobiota)* accounted for up to 35% in association with *Navicula* sp., not being found in other assemblages.

The assemblages of *T. indica* differed from other MS cultures in poor diversity of classes *Alphaproteobacteria* and *Gammaproteobacteria*, represented by single OTUs (Table S2). As mentioned above, such a sharp decrease in taxonomic richness is probably due to the numerous washings of algal cells during isolation of the monoalgal cultures. As a result, in all MS assemblages of *T. indica*, one OTU assigned to genus *Marivibrio* prevailed, reaching proportions of 98.0–99.9% in three samples and 30.2% in the fourth one, where genus *Alloalcanivorax* prevailed (Table S2). Interestingly, single *Marivibrio*-related sequences were detected in the MS natural communities. In our opinion, this phenomenon may be determined by a tight attachment of *Marivibrio* to the surface of algal cells.

### 3.8. Correlation between Microalgae Species and Taxonomic Composition of Prokaryotic Assembleges

The Mantel test was additionally applied to assess the correlation between the microalgae species and the relative abundance of prokaryotic genera. Each microalgal species from both EPS and MS assemblages correlated significantly with some associated prokaryotic genera. 

**Figure 7:**
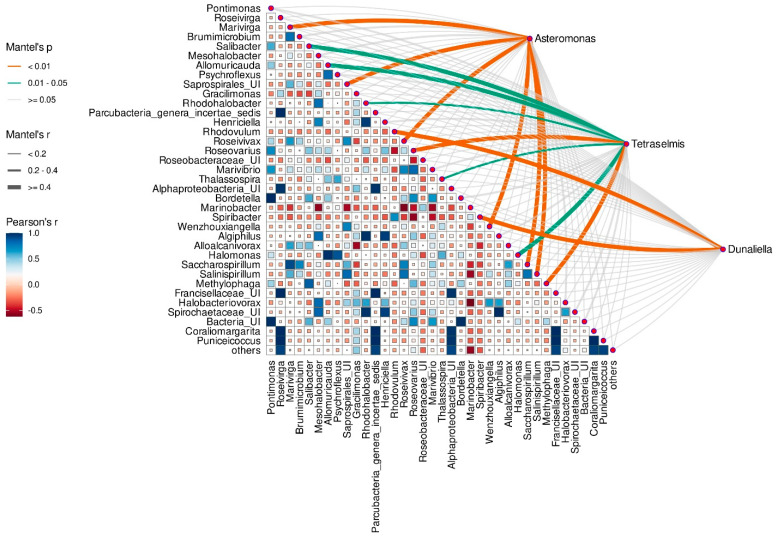
Mantel’s test showing Spearman’s correlations between the microalga species and the relative abundance of prokaryotic genera from microbiomes of the EPS monoalgal cultures. The heat map shows Pearson’s correlations between abundances of prokaryotes. Edge width, size and color of block denote the Mantel *r* statistic, whereas edge color denotes the Mantel *p* value based on 9999 permutations.

*A. gracilis* had significant correlations with genera *Marivirga*, *Roseivivax*, *Wenzhouxiangella*, *Salinispirillum*, *Saccharospirillum*, and unidentified *Saprospirales* (Figure 7). *Dunaliella* sp. correlated significantly with abundant Alpha- and Gammaproteobacteria, *Rhodovulum* and *Spiribacter*, respectively. Genera *Roseovarius* and *Methylophaga* (Mantel *p* value < 0.01), *Salibacter*, *Allomuricauda*, and *Halomonas* (Mantel *p* value < 0.05) were strongly associated with *T. indica*, whereas *Rhodohalobacter* and *Thalassospira* correlated with this alga moderately (Figure 7).

**Figure 8:**
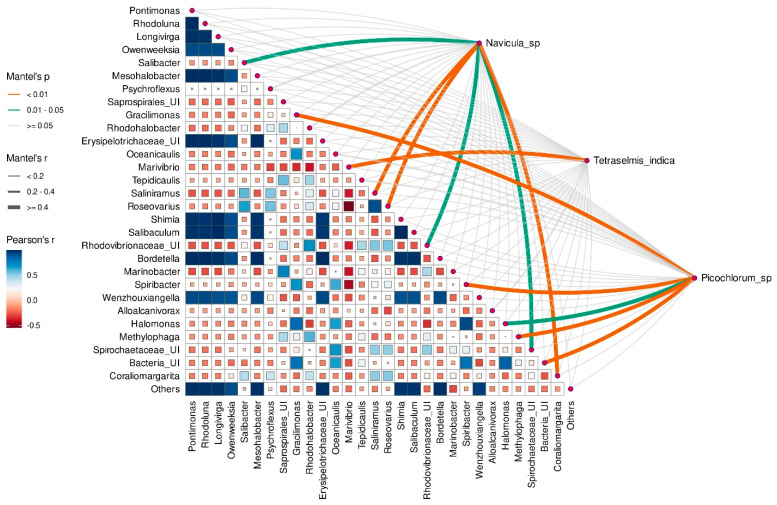
Mantel’s test showing Spearman’s correlations between the microalga species and the relative abundance of prokaryotic genera from microbiomes of the MS monoalgal cultures. The heat map shows Pearson’s correlations between abundances of prokaryotes. Edge width, size and color of block denote the Mantel *r* statistic, whereas edge color denotes the Mantel *p* value based on 9999 permutations.

*Navicula* spp. correlated significantly with *Roseovarius*, *Saliniramus*, *Coraliomargarita* (Mantel *p* value < 0.01), *Salibacter*, unidentified *Rhodovibrionaceae* and *Spirochaetaceae* (Mantel *p* value < 0.05) (Figure 8). Genera *Gracilimonas*, *Spiribacter*, *Methylophaga* (Mantel *p* value < 0.01), and *Halomonas* (Mantel *p* value < 0.05) were strongly associated with *Picochlorum* sp., whereas *Marivibrio* greatly correlated with *T. indica* (Figure 8).

## 4. Discussion

In this study, we have analyzed the microbiomes of monoalgal cultures obtained from inland saline waters using DNA metabarcoding, and compared them to natural prokaryotic communities. The monoalgal cultures were isolated from two sites near Elton Lake with salinity levels of 100–110‰. It is worth noting that interactions between microalgae and bacterial communities under intermediate salinity are less studied than in marine [15,20,38,39] or at near-saturated salinities [40,41]. Moreover, to date, few studies have focused on changes in prokaryotic communities associated with halophilic microalgae upon their transfer from environment to laboratory cultures. For this study, dominant microalgae were taken including green algae *Dunaliella* sp., *T. indica*, and *A. gracilis* in the ephemeral pond near Solyanka River (EPS) and diatom *Navicula* sp., green algae *Picochlorum* sp., and *T. indica* in Malaya Smorogda River (MS). Results of sequencing the V4 region of the 18S rRNA gene convincingly confirmed the microscopic identification of the isolated cultures. All these taxa are halophilic and have been previously isolated from hypersaline water bodies [24,42,43,44,45]. Previously, *Dunaliella* sp., *Picochlorum* sp., and *T. indica* have been detected by NGS in saline rivers of the Elton Lake basin [27]. However, their cultures were isolated from these habitats for the first time in this study. *A. gracilis* and *Navicula salinicola* have not been previously recorded there. Given their potential as promising sources of carotenoids, biodiesel products, and polyunsaturated fatty acids [25,46,47,48,49], studying prokaryotic assemblages associated with these microalgae is of particular importance.

In both natural communities and microalgae cultures, the most abundant phylotypes belonged to the bacterial genera common in habitats with intermediate salinity [50], and were represented by moderately halophilic bacteria [51]. The taxonomic richness of bacteria in the laboratory monoalgal cultures was significantly lower than in natural communities, which is consistent with literature data on the change in microalgae-associated bacterial communities upon transition to laboratory cultivation [38]. However, a decrease in the prokaryotic richness and diversity was much more pronounced in the control communities without microalgae. Moreover, most control samples did not produce PCR amplicons at all, which can be explained by the attenuation and cessation of prokaryotic growth in the minimal mineral medium in the absence of microalgae acting as the primary producer of organic matter. It should be noted that diversity indices remained quite high in most microalgae cultures except for those washed many times during the isolation procedure. Algae likely contribute to the maintenance of sufficiently diverse, balanced, and even prokaryotic assemblages in laboratory cultures.

At the level of high-ranking taxa, classes *Alphaproteobacteria* and *Gammaproteobacteria* of phylum *Pseudomonadota*, as well as phylum *Bacteroidota,* turned out to be dominant groups in the microalgae cultures of all species, despite significant differences between the taxonomic composition of EPS and MS natural samples. The predominance of these taxa in microalgae associations has been described previously in both natural and pilot cultures [52]. Thus, it was shown that *Pseudomonadota* and *Bacteroidota* dominated in all samples of marine communities associated with *Haematococcus lacustris,* despite the pronounced difference in environmental conditions and bacterial diversity [38]. Furthermore, the predominance of *Bacteroidota* and *Pseudomonadota* has reached up to 90% in bacterial communities associated with laboratory cultures of the biofuel-producing green microalgae *Nannochloropsis* [52] and in all industrial algae production systems [53]. Both of the two prevalent bacterial phyla, *Proteobacteria* and *Bacteroidota*, are capable of rapidly decomposing complex organic matter, facilitating a direct transfer of organic carbon from algae to more demanding prokaryotes [54,55]. Our data demonstrated that phylum *Balneolota* also dominated in bacterial assemblages associated with halophilic microalgae, reaching 25–28%. Its prevalence is determined mainly by the development of *Balneolaceae* family members, which include bacteria growing aerobically at 5–10% NaCl [51]. Although there is no information on the dominance of this group in assemblages of halophilic microalgae, one member of the phylum *Balneolota* was shown to be adapted to a cyanobacteria-associated lifestyle in a hypersaline soda lake [56]. However, in the *Tetraselmis indica* culture, freed from most free-living bacteria as a result of numerous washings of individual algal cells, *Bacteroidota* and *Balneolota* were absent completely. This indicates their likely free-living state, not being attached to the surface of microalgal cells. This suggestion is consistent with data demonstrating that *Bacteroidota* are the most abundant in free-living bacterial communities associated with microalgae regardless of the kind of alga, whereas *Pseudomonadota* have been dominant in the cell-attached fractions of algal cultures [57]. In addition to the similarity in taxonomic composition at the level of high-ranking taxa, differences depending on the taxonomic position of microalgae were revealed. Namely, phylum *Verrucomicrobiota* was detected only in the assemblages of diatoms, and its relative abundance reached 35%. This fact can probably be related to the ability of free-living *Verrucomicrobiota* to consume fucose- and rhamnose-containing sulfated polysaccharides produced by diatoms, which are difficult for other bacteria to decompose [58].

Analysis of the taxonomic composition of prokaryotic assemblages at the family and genus levels revealed common, occasional, and species-specific taxa of bacteria associated with different microalgae species. The distribution of most *Bacteroidota* representatives in microalgae cultures was occasional, probably due to a similar function of different taxa, consuming high-molecular dissolved organic matter and providing low-molecular products to microalgae. Some species of the dominant genus *Psychroflexus* have been previously found and isolated from associations with algae, and their dependence on algal cells due to epiphytism has been described [59]. For another species, *Mesohalobacter halotolerans*, porphyrin and chlorophyll metabolism has been predicted [50]. Some dominated phylotypes were related to the family *Cryomorphaceae*, which has been associated with phytoplankton blooms [60]. Moreover, some representatives of the family were isolated from assemblages of microalgae [61]. The presence of unidentified *Saprospiraceae* only in association with *Navicula* sp. and *Picochlorum* sp. may indicate their specificity to respective algal taxa. In addition, members of the family *Cryomorphaceae* are known to be largely associated with marine sediments and eukaryotes, and are not free-living organisms. This likely reflects their propensity to attach to surfaces, as well as their ability to degrade associated complex nutrient sources [62,63] and cause lysis of the diatom *Chaetoceros ceratosporum* [53,64,65]. Representatives of another family, *Saprospiraceae,* have been described in 100% of samples from an industrial production system of *Nannochloropsis salina* [53]. 

Phylotypes of *Balneolaceae* that dominated all monoalgal cultures were assigned to two genera, *Rhodohalobacter* and *Gracilimonas*, regardless of the microalga species. Some representatives of *Gracilimonas* have been previously isolated from cyanobacterial culture *Synechococcus* and from seaweed [66,67]. *Rhodohalobacter mucosus* is able to hydrolyze starch, alginate, casein, and other complex organic substances produced by algae [68]. 

*Alphaproteobacteria* and *Gammaproteobacteria* were the most abundant and diverse prokaryotes, and also included specific complexes of genera for the microalgae species (species-specific complexes): *Roseivivax* for *A. gracilis*; *Rhodovulum* for *Dunaliella* sp.; *Rhodovulum, Thalassospira,* and *Marivibrio* for *T. indica*; and *Roseovarius, Saliniramus,* and unidentified *Rhodovibrionaceae* for *Navicula* spp. This fact indicates the significance of these bacterial taxa for the growth of the microalgae. Among *Alphaproteobacteria,* the phylotypes belonging to family *Rhodobacteraceae* were the most abundant and were present in all microalgae cultures, except for *T. indica* washed out from free-living bacterial associates. The presence of *Rhodobacteraceae* in the phycosphere of marine diatoms is well known and may be explained by the ability of these Alphaproteobacteria to degrade NH_3_-containing compatible solutes, such as methylamines and glycine betaine, releasing ammonium as a nitrogen source for some diatoms [69]. Moreover, some members of this family have been shown to possess the complete adenosylcobalamin (vitamin B12) pathway, and therefore they may be potential suppliers of B12 to microalgae, providing a beneficial effect on their long-term survival [39]. In addition, bacteria of the *Roseobacter* clade can supply microalgae with plant growth promoters [70], thereby exerting a positive effect on growth [71] and ensuring proper algal morphogenesis [72]. A significant proportion of unidentified sequences belonging to families *Rhodobacteraceae* and *Rhodovibrionaceae* opens up the prospect of searching for new bacterial taxa in these assemblages of the halophilic alga. 

In addition, it is important to emphasize that in the monoalgal cultures of *T. indica,* washed many times from accompanied bacteria, only one alphaproteobacterial phylotype belonging to genus *Marivibrio* was an absolute dominant. The possibility of epiphytic growth of *Marivibrio* has not been described yet, since the representatives of this monospecific genus had been isolated from underground rock salt [73]. In contrast, adhesion of Alphaproteobacteria belonging to genus *Ruegeria* to the cell surface of *T. indica* has been previously shown [74].

Genus *Marinobacter*, a representative of class *Gammaproteobacteria*, was the most frequently occurring dominant in the monoalgal assemblages. Its relative abundance increased significantly upon transition of the algae to laboratory cultivation, but the highest proportion of *Marinobacter* was noted in the alga-free controls, which demonstrates an ability of this oligotrophic bacterium to survive under starvation. At the same time, *Marinobacter adhaerens* is known to stimulate the growth of various diatoms including *Thalassiosira weissflogii* [71,75,76]. Genus *Marinobacter* also dominated in the studied assemblages of green halophilic microalgae. A necessity of *Marinobacter* for microalgae may be related to the production and release of Fe-chelating siderophores, which enhance algal production [77]. Genus *Spiribacter* was another common dominant for all studied microalgal cultures. The relative abundance of its phylotypes was significant both in the natural communities and in assemblages of the microalga cultures. This is not surprising, because *Spiribacter* is a widespread planktonic moderately halophilic bacterium [78]. However, the highest relative abundances of *Spiribacter* were recorded in the assemblages of *Dunaliella* sp., a well-known halophilic microalga, due to its ability to produce significant amounts of glycerol as a compatible solute [40]. In turn, many *Spiribacter* species are able to catabolically degrade glycerol [79].

The presence of genus *Methylophaga* among the dominants common to all cultures most likely indicates its role as a symbiont of halophilic microalgae. The mutual trophic relationship is probably determined by the ability of these bacteria to use the methylation metabolites (such as trimethylamines, dimethylsulfopropionate and dimethylsulfide) produced by algae as carbon and energy sources. On the other hand, it is known that some strains of halophilic methylobacteria isolated from associations with algae produce auxins (indole-3-acetic acid) [80]. In addition, for the strain *Methylophaga* sp. isolated from the association with *Microchloropsis salina*, synthesis of vitamins B1 (thiamine), B7 (biotin), and B12 (cobalamin) has been predicted [81]. Findings of sequences assigned to the genus of hydrocarbon-degrading bacteria *Alloalcanivorax* in the microalgae assemblages are consistent with current observations. Previously, *Alcanivorax borkumensis* has been isolated from cultures of dinoflagellate *Gymnodinium catenatum* [82]. However, according to our data, *Alloalcanivorax* are not obligatory in the associations with the halophilic microalgae unlike *Marinobacter*. The specificity of particular bacterial associates for a certain algal species can be demonstrated using assemblages of *A. gracilis* cultures, where genera *Salinispirillum* and *Saccharospirillum* were found, in contrast to assemblages of other algae where these bacteria were absent. Representatives of these genera are able to form specific mutual trophic relationships with microalgae like the bacterial taxa described above [83,84]. *Coraliomargarita* belonging to phylum *Verrucomicrobiota* is another crucial bacterial taxon, which is specific for assemblages of *Navicula* sp., where its relative abundance reached 35%. At the same time, it was absent in all other algal assemblages. This genus has been previously found in association with the ichthyotoxic dinoflagellate *Cochlodinium (Margalefidinium) polykrikoides*, which, under bloom conditions, release complex high-molecular organic compounds serving as a preferential carbon and energy source for *Coraliomargarita* [85].

## 5. Conclusions

In conclusion, the following statements are formulated: (i) Prokaryotic assemblages of halophilic microalgae change considerably upon transition to laboratory cultivation. Microalgae support rather diverse and even assemblages of prokaryotes despite the disappearance of a significant number of taxa. (ii) Classes *Alphaproteobacteria* and *Gammaproteobacteria*, phyla *Balneolota* and *Bacteroidota,* are the most considerable dominants regardless of algal taxonomic affiliation and site of isolation. (iii) Despite a certain similarity of prokaryotic assemblages, microalgal species feature taxon-specific complexes of dominant prokaryotic genera as a result of selective advantages during cultivation. The *Alphaprotheobacteria* class contains the largest number of taxon-specific genera, likely due to their close association with the microalgae. (iv) The distribution of most *Bacteroidota* genera in microalgal assemblages is random except for unidentified *Saprospirales*, which reveal a strong association with *A. gracilis.* (v) Alphaproteobacteria of the *Marivibrio* genus became dominant in association with *T. indica* being cleared from most free-living prokaryotes, likely due to a firm attachment to the microalgae cells.

Our results provide insights into the structure and functioning of halophilic microbial communities formed by microalgae and prokaryotes. They may be useful for further developing cultivation and control systems applied for biotechnologies of halophilic algae. On the other hand, the data will contribute to the development of approaches for obtaining new prokaryotes with a microalgae-associated lifestyle.

## Figures and Tables

**Table 1:** Taxonomy of monoalgal cultures isolated from hypersaline sites and their closest homologues in 18S rRNA gene sequences from database GenBank NCBI.

OTU (NCBI Accession No.)	Closest Homologue (NCBI Accession No.)	Query Cover (%)	Similarity (%)	Identified as (Number of Cultures)	Taxonomy (Phylum, Class)	Sampling Site
19Z-93_132-05-7 (OR037277)	*Dunaliella* sp. (MN907401)	100	99.76	*Dunaliella* sp. (4)	Chlorophyta, Chlorophyceae	EPS
19Z-93_132-05-9 (OR037278)	*Navicula salinicola* (MT012298)	100	99.28	*Navicula* sp. 1 (2)	Bacillariophyta, Bacillariophyceae	MS
19Z-93_132-05-24 (OR037279)	*Navicula salinicola* (MT012298)	100	98.89	*Navicula* sp. 2 (3)	Bacillariophyta, Bacillariophyceae	MS
19Z-93_132-05-1 (OR037280)	*Tetraselmis indica* (HQ651184)	100	99.76	*Tetraselmis indica* (4)	Chlorophyta, Chlorodendrophyceae	EPS
19Z-93_132-05-1 (OR037280)	*Tetraselmis indica* (HQ651184)	100	99.76	*Tetraselmis indica* (4)	Chlorophyta, Chlorodendrophyceae	MS
19Z-93_132-05-3 (OR037281)	*Picochlorum* sp. (MK973100)	100	99.76	*Picochlorum* sp. (5)	Chlorophyta, Trebouxiophyceae	MS
19Z-93_132-05-2 (OR037282)	*Asteromonas gracilis* (JN033244)	100	99.76%	*Asteromonas gracilis* (4)	Chlorophyta, Chlorophyceae	EPS

## Data Availability

The raw sequence reads were deposited into the Sequence Read Archive (SRA) of NCBI under the accession numbers SRX25767012-SRX25767043. The Bioproject accession number is PRJNA1149946. The partial 18S rRNA genes of microalgae obtained in this study were deposited to the GenBank (NCBI) under accession numbers OR037277-OR037282.

## Supplementary Materials

The following supporting information can be downloaded at https://www.mdpi.com/article/10.3390/microorganisms12101947/s1, Figure S1: Dendrogram based on the Bray–Curtis metrics of the prokaryotic assemblages associated with cultures of halophilic microalgae, derived from the ephemeral pond near Solyanka River; Figure S2: Dendrogram based on the Bray–Curtis metrics of the prokaryotic assemblages associated with cultures of halophilic microalgae, derived from Malaya Smorogda River; Table S1: Relative abundances of dominant genera (more than 1% of reads in a sample) in the prokaryotic assemblages from the monoalgal and control (without algae) cultures from the ephemeral pond near Solyanka River; Table S2: Relative abundances of dominant genera (more than 1% of reads in a sample) in the prokaryotic assemblages from the monoalgal and control (without algae) cultures from Malaya Smorogda River; Table S3: Numbers of raw reads, high-quality reads, and taxonomic richness in DNA libraries from the prokaryotic community of the ephemeral pond near Solyanka River and the prokaryotic assemblages associated with the derived monoalgal cultures; Table S4: Numbers of raw reads, high-quality reads, and taxonomic richness in DNA libraries from the prokaryotic community of Malaya Smorogda River and the prokaryotic assemblages associated with the derived monoalgal cultures.

## Abbreviations

MS, Malaya Smorogda River; EPS, ephemeral pond near Solyanka River; OTU, operational taxonomic unit.
