# Supplementary material for: The Cultivation of Halophilic Microalgae Shapes the Structure of Their Prokaryotic Assemblages
Source: Microorganisms. 2024 Sep 26;12(10):1947. doi: 10.3390/microorganisms12101947 (PMC11509377; doi:10.3390/microorganisms12101947)
Supplement: Supplementary file 1 [file microorganisms-12-01947-s001.zip › microorganisms-3208837-supplementary.docx]

**Supplementary Figures S1-S2, Tables S1-S4**

*Article*

**Cultivation of the halophilic microalgae shapes a structure of their prokaryotic assemblages**

**Elena A. Selivanova ^1,^*, Michail M. Yakimov ^2^, Vladimir Y. Kataev ^1^, Yuri А. Khlopko ^1^, Alexander S. Balkin ^1^** **and Andrey O. Plotnikov ^1,^***

^1^ Institute for Cellular and Intracellular Symbiosis of the Ural Branch of the Russian Academy of Sciences, Orenburg Federal Research Center of the Ural Branch of the Russian Academy of Sciences, Orenburg, Russia; [vladimir0334@yandex.ru](mailto:vladimir0334@yandex.ru) (V.Y.K.), [140374@mail.ru](mailto:140374@mail.ru) (Y.А.K.), [balkinas@yandex.ru](mailto:balkinas@yandex.ru) (A.S.B.)

^2^ Extreme Microbiology, Biotechnology and Astrobiology Group, Institute of Polar Research, ISP-CNR, Messina, Italy; mikhail.iakimov@cnr.it

***** Correspondence: [selivanova-81@mail.ru](mailto:selivanova-81@mail.ru) (E.A.S.); [protoz@mail.ru](mailto:protoz@mail.ru) (A.O.P.); Tel.: +73532775417

**Figure S1.** Dendrogram based on the Bray-Curtis metrics of the prokaryotic assemblages associated with cultures of halophilic microalgae, derived from the ephemeral pond near the Solyanka River.


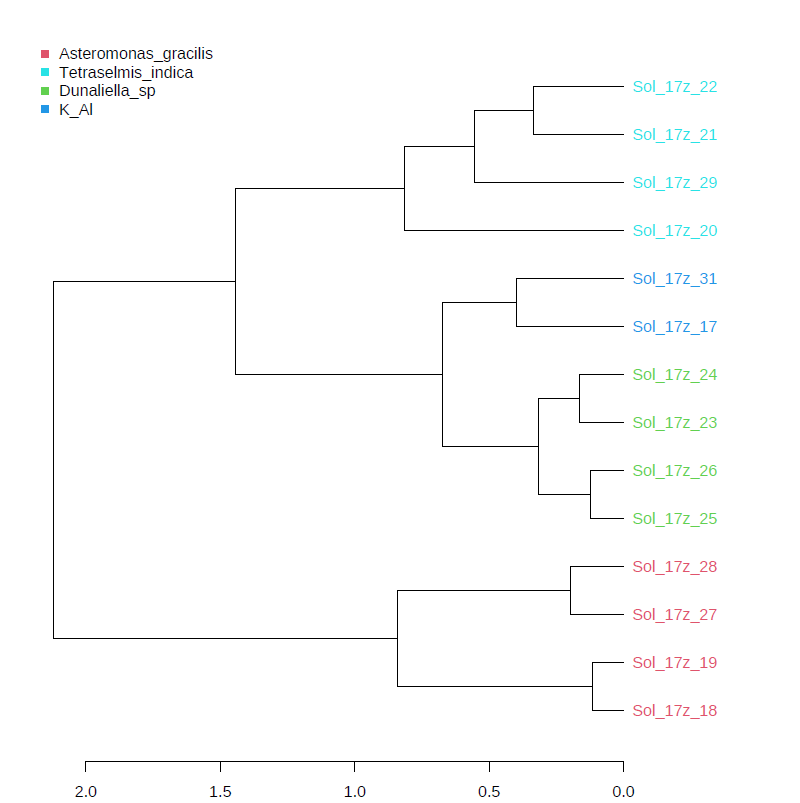


**Figure S2.** Dendrogram based on the Bray-Curtis metrics of the prokaryotic assemblages associated with cultures of halophilic microalgae, derived from the Malaya Smorogda River.

**
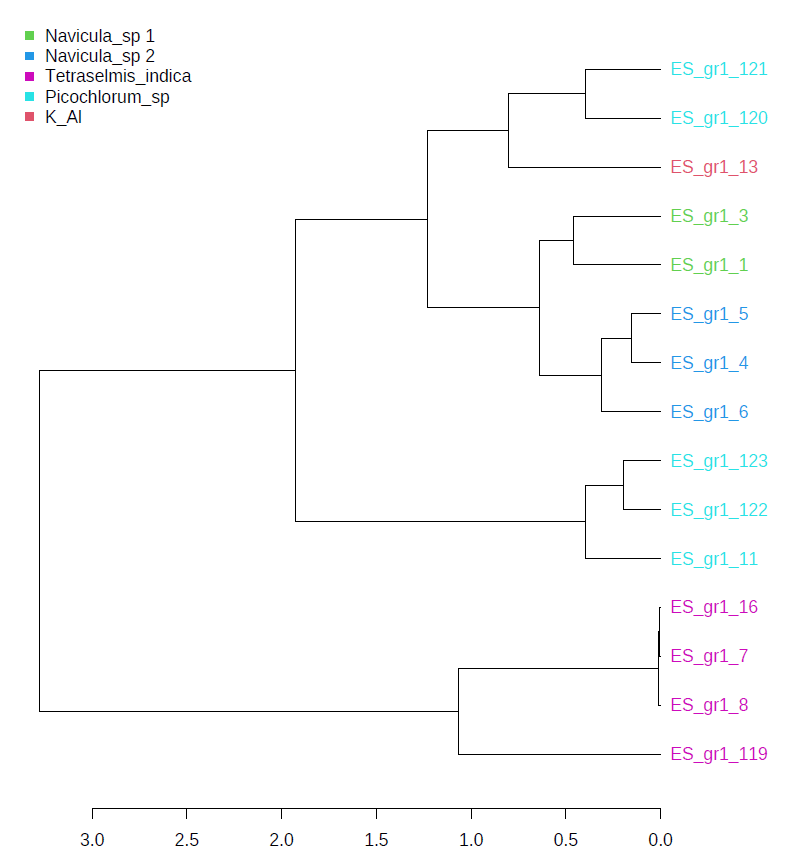
**

**Table S1.** Relative abundances of dominant genera (more than 1% of reads in a sample) in the prokaryotic assemblages from the monoalgal and control (without algae) cultures from the ephemeral pond near the Solyanka River. UI – unidentified.

| **Taxonomy of dominant prokaryotic genera** | | | **Natural sample** | **Algal cultures** | | | | | | | | | | | | **Control cultures** | |
| --- | --- | --- | --- | --- | --- | --- | --- | --- | --- | --- | --- | --- | --- | --- | --- | --- | --- |
| **class** | **family** | **genus** |  | ***Asteromonas gracilis*** | | | | ***Tetraselmis indica*** | | | | ***Dunaliella* sp.** | | | |  |  |
| *Actinomycetes* | *Microbacteriaceae* | *Pontimonas* | 0,0 | 0,0 | 0,0 | 0,0 | 0,0 | 0,0 | 0,0 | 0,0 | 1,4 | 0,0 | 0,0 | 0,0 | 0,0 | 0,0 | 0,0 |
| *Cytophagia* | *Roseivirgaceae* | *Roseivirga* | 1,2 | 0,0 | 0,0 | 0,0 | 0,0 | 0,0 | 0,0 | 0,0 | 0,0 | 0,0 | 0,0 | 0,0 | 0,0 | 0,0 | 0,0 |
|  | *Marivirgaceae* | *Marivirga* | 0,0 | 0,1 | 0,1 | 0,8 | 2,2 | 0,2 | 0,0 | 0,0 | 0,0 | 0,0 | 0,0 | 0,0 | 0,0 | 0,0 | 0,0 |
| *Flavobacteriia* | *Crocinitomicaceae* | *Brumimicrobium* | 0,0 | 0,0 | 0,0 | 1,2 | 3,5 | 0,2 | 0,0 | 0,0 | 0,0 | 0,0 | 0,0 | 0,0 | 0,0 | 0,0 | 2,5 |
|  | *Salibacteraceae* | *Salibacter* | 0,0 | 0,0 | 0,2 | 0,0 | 0,0 | 0,0 | 0,0 | 8,6 | 7,1 | 0,8 | 0,0 | 0,3 | 0,0 | 0,0 | 0,0 |
|  | *Flavobacteriaceae* | *Mesohalobacter* | 0,7 | 0,9 | 3,1 | 0,8 | 2,1 | 5,6 | 0,0 | 0,0 | 0,0 | 0,2 | 0,0 | 0,0 | 0,0 | 0,0 | 0,0 |
|  |  | *Allomuricauda* | 0,0 | 0,0 | 0,0 | 0,0 | 0,0 | 0,0 | 1,6 | 0,0 | 1,0 | 0,0 | 0,0 | 0,0 | 0,0 | 0,0 | 0,2 |
|  |  | *Psychroflexus* | 0,2 | 0,5 | 1,1 | 0,0 | 0,0 | 0,0 | 18,8 | 0,0 | 1,6 | 0,0 | 4,8 | 0,0 | 0,0 | 2,2 | 0,3 |
| *Saprospiria* | *Saprospirales* UI | | 0,0 | 13,0 | 10,3 | 8,9 | 10,8 | 0,0 | 0,0 | 0,0 | 0,0 | 0,0 | 0,0 | 0,0 | 0,0 | 2,2 | 0,0 |
| *Balneolia* | *Balneolaceae* | *Gracilimonas* | 5,4 | 3,5 | 3,9 | 0,3 | 0,6 | 5,2 | 2,7 | 0,6 | 0,9 | 2,5 | 5,9 | 1,0 | 1,1 | 1,4 | 1,7 |
|  |  | *Rhodohalobacter* | 0,4 | 1,9 | 1,9 | 0,8 | 1,7 | 23,3 | 3,9 | 0,5 | 1,7 | 0,5 | 0,8 | 1,1 | 3,5 | 1,3 | 1,7 |
| *"Candidatus* Paceibacterota*"* | | | 22,6 | 0,0 | 0,0 | 0,0 | 0,0 | 0,0 | 0,0 | 0,0 | 0,0 | 0,0 | 0,0 | 0,0 | 0,0 | 0,0 | 0,0 |
| *Alphaproteobacteria* | *Hyphomonadaceae* | *Henriciella* | 0,1 | 0,0 | 0,1 | 0,0 | 0,0 | 1,1 | 0,2 | 0,0 | 0,1 | 0,0 | 0,0 | 0,0 | 0,0 | 0,1 | 0,3 |
|  | *Paracoccaceae* | *Rhodovulum* | 0,0 | 5,5 | 4,8 | 2,0 | 1,0 | 0,4 | 0,1 | 0,1 | 0,0 | 21,5 | 9,3 | 7,5 | 3,8 | 0,0 | 0,0 |
|  | *Roseobacteraceae* | *Roseivivax* | 0,0 | 4,6 | 4,6 | 9,6 | 8,6 | 0,6 | 0,6 | 0,0 | 7,9 | 0,0 | 0,0 | 0,0 | 0,0 | 0,0 | 0,0 |
|  |  | *Roseovarius* | 10,5 | 5,0 | 4,7 | 7,8 | 8,3 | 20,8 | 8,6 | 9,7 | 26,9 | 0,8 | 1,5 | 0,2 | 0,7 | 0,9 | 5,5 |
|  |  | *Roseobacteraceae* UI | 1,1 | 4,4 | 3,1 | 3,8 | 5,4 | 0,6 | 0,6 | 2,5 | 1,8 | 4,3 | 5,4 | 0,8 | 3,2 | 11,6 | 4,9 |
|  | *Rhodospirillaceae* | *Marivibrio* | 0,5 | 0,2 | 0,4 | 2,9 | 2,0 | 2,7 | 0,5 | 0,0 | 4,2 | 0,0 | 0,0 | 0,0 | 0,0 | 0,0 | 0,0 |
|  | *Thalassospiraceae* | *Thalassospira* | 0,0 | 7,1 | 6,4 | 1,0 | 1,2 | 1,3 | 12,9 | 9,5 | 1,0 | 0,0 | 0,0 | 0,0 | 0,0 | 0,0 | 0,0 |
|  | *Alphaproteobacteria* UI | | 11,7 | 0,0 | 0,0 | 0,0 | 0,0 | 2,4 | 0,0 | 0,0 | 0,0 | 0,0 | 0,0 | 0,0 | 0,0 | 0,0 | 0,0 |
| *Betaproteobacteria* | *Alcaligenaceae* | *Bordetella* | 0,0 | 0,0 | 0,0 | 0,0 | 0,0 | 0,0 | 0,0 | 0,0 | 1,0 | 0,0 | 0,0 | 0,0 | 0,0 | 0,0 | 0,0 |
| *Gammaproteobacteria* | *Marinobacteraceae* | *Marinobacter* | 0,2 | 1,9 | 8,9 | 2,2 | 2,6 | 2,7 | 37,8 | 40,0 | 16,4 | 28,5 | 34,3 | 52,4 | 45,3 | 79,9 | 51,5 |
|  | *Ectothiorhodospiraceae* | *Spiribacter* | 0,0 | 16,4 | 16,0 | 0,8 | 1,3 | 1,8 | 2,4 | 4,3 | 6,9 | 34,9 | 34,3 | 35,9 | 40,5 | 0,0 | 19,6 |
|  | *Wenzhouxiangellaceae* | *Wenzhouxiangella* | 0,1 | 3,5 | 3,1 | 0,1 | 0,1 | 0,0 | 0,0 | 0,0 | 0,0 | 0,0 | 0,0 | 0,0 | 0,0 | 0,0 | 0,0 |
|  | *Algiphilaceae* | *Algiphilus* | 0,0 | 0,0 | 0,0 | 0,0 | 0,0 | 1,2 | 0,0 | 0,0 | 0,0 | 0,0 | 0,0 | 0,0 | 0,0 | 0,0 | 0,0 |
|  | *Alcanivoracaceae* | *Alloalcanivorax* | 0,0 | 0,1 | 0,1 | 1,1 | 1,7 | 0,0 | 0,1 | 2,0 | 0,5 | 0,0 | 0,0 | 0,0 | 0,0 | 0,0 | 0,0 |
|  | *Halomonadaceae* | *Halomonas* | 0,0 | 0,0 | 0,0 | 0,0 | 0,0 | 0,0 | 2,0 | 0,0 | 0,9 | 0,0 | 0,0 | 0,0 | 0,0 | 0,0 | 0,0 |
|  | *Saccharospirillaceae* | *Saccharospirillum* | 0,0 | 0,6 | 0,4 | 19,4 | 22,7 | 1,9 | 0,0 | 0,0 | 0,0 | 0,0 | 0,0 | 0,0 | 0,0 | 0,0 | 0,0 |
|  |  | *Salinispirillum* | 0,3 | 13,8 | 12,3 | 31,8 | 15,9 | 0,0 | 0,0 | 0,0 | 0,0 | 0,4 | 0,0 | 0,0 | 0,0 | 0,0 | 0,0 |
|  | *Piscirickettsiaceae* | *Methylophaga* | 0,2 | 2,2 | 2,1 | 1,6 | 2,2 | 6,8 | 5,6 | 20,3 | 14,7 | 3,5 | 1,1 | 0,3 | 0,7 | 0,0 | 11,1 |
|  | *Francisellaceae* | *Francisellaceae* UI | 25,5 | 0,0 | 0,0 | 0,0 | 0,0 | 0,0 | 0,0 | 0,0 | 0,0 | 0,0 | 0,0 | 0,0 | 0,0 | 0,0 | 0,0 |
| *Bacteriovoracia* | *Halobacteriovoraceae* | *Halobacteriovorax* | 5,2 | 12,5 | 9,7 | 0,7 | 2,9 | 14,8 | 0,0 | 0,0 | 0,0 | 0,0 | 0,0 | 0,0 | 0,0 | 0,0 | 0,0 |
| *Spirochaetia* | *Spirochaetaceae* | *Spirochaetaceae* UI | 0,0 | 0,0 | 0,0 | 0,1 | 0,2 | 4,4 | 0,0 | 0,0 | 0,0 | 0,0 | 0,0 | 0,0 | 0,0 | 0,0 | 0,0 |
| *Bacteria* UI | | | 0,1 | 0,0 | 0,0 | 0,0 | 0,0 | 0,0 | 0,0 | 0,0 | 1,0 | 0,0 | 0,0 | 0,0 | 0,0 | 0,0 | 0,0 |
| *Opitutia* | *Coraliomargaritaceae* | *Coraliomargarita* | 1,6 | 0,0 | 0,0 | 0,0 | 0,0 | 0,0 | 0,0 | 0,0 | 0,0 | 0,0 | 0,0 | 0,0 | 0,0 | 0,0 | 0,0 |
|  | *Puniceicoccaceae* | *Puniceicoccus* | 1,4 | 0,0 | 0,0 | 0,0 | 0,0 | 0,0 | 0,0 | 0,0 | 0,0 | 0,0 | 0,0 | 0,0 | 0,0 | 0,0 | 0,0 |
| others | | | 10,8 | 2,2 | 2,8 | 2,4 | 2,8 | 2,0 | 1,7 | 1,9 | 2,9 | 2,2 | 2,5 | 0,4 | 1,1 | 0,4 | 0,8 |

**Table S2.** Relative abundances of dominant genera (more than 1% of reads in a sample) in the prokaryotic assemblages from the monoalgal and control (without algae) cultures from the Malaya Samoroda River. UI – unidentified.

| **Taxonomy of dominant prokaryotic genera** | | | | **Natural samples** | | | | **Algal cultures** | | | | | | | | | | | | | | | | | | | | | | | | | | | | **Control culture** |
| --- | --- | --- | --- | --- | --- | --- | --- | --- | --- | --- | --- | --- | --- | --- | --- | --- | --- | --- | --- | --- | --- | --- | --- | --- | --- | --- | --- | --- | --- | --- | --- | --- | --- | --- | --- | --- |
| **class** | **family** | **genus** |  | | | | ***Navicula* sp.1** | | | | ***Navicula* sp.2** | | | | | | ***Tetraselmis indica*** | | | | | | | | ***Picochlorum* sp.** | | | | | | | | | |  | |
| *Actinomycetes* | *Microbacteriaceae* | *Pontimonas* | 2,8 | | 2,9 | | 0,0 | | 0,0 | | 0,0 | | 0,0 | | 0,0 | | 0,0 | | 0,0 | | 0,0 | | 0,0 | | 0,0 | | 0,0 | | 0,0 | | 0,0 | | 0,0 | | 0,0 | |
|  |  | *Rhodoluna* | 27,9 | | 25,3 | | 0,0 | | 0,0 | | 0,0 | | 0,0 | | 0,0 | | 0,0 | | 0,0 | | 0,0 | | 0,0 | | 0,0 | | 0,0 | | 0,0 | | 0,0 | | 0,0 | | 0,0 | |
|  | *Sporichthyaceae* | *Longivirga* | 5,0 | | 6,5 | | 0,0 | | 0,0 | | 0,0 | | 0,0 | | 0,0 | | 0,0 | | 0,0 | | 0,0 | | 0,0 | | 0,0 | | 0,0 | | 0,0 | | 0,0 | | 0,0 | | 0,0 | |
| *Flavobacteriia* | *Owenweeksiaceae* | *Owenweeksia* | 2,4 | | 2,6 | | 0,0 | | 1,4 | | 0,0 | | 0,0 | | 0,0 | | 0,0 | | 0,0 | | 0,0 | | 0,0 | | 0,0 | | 0,0 | | 0,0 | | 0,0 | | 0,0 | | 0,0 | |
|  | *Salibacteraceae* | *Salibacter* | 0,0 | | 0,1 | | 0,0 | | 0,0 | | 6,2 | | 5,8 | | 0,0 | | 0,0 | | 0,0 | | 0,0 | | 0,0 | | 0,0 | | 0,0 | | 0,0 | | 0,0 | | 0,0 | | 0,0 | |
|  | *Flavobacteriaceae* | *Mesohalobacter* | 1,7 | | 1,6 | | 0,0 | | 0,0 | | 0,0 | | 0,0 | | 0,0 | | 0,0 | | 0,0 | | 0,0 | | 0,0 | | 0,0 | | 0,0 | | 0,0 | | 0,0 | | 0,0 | | 0,0 | |
|  |  | *Psychroflexus* | 3,3 | | 3,4 | | 0,0 | | 3,3 | | 3,9 | | 8,0 | | 18,3 | | 0,0 | | 0,0 | | 0,0 | | 0,0 | | 0,0 | | 0,0 | | 0,1 | | 0,2 | | 13,2 | | 0,0 | |
| *Saprospiria* | *Saprospirales* UI | | | 0,0 | | 0,0 | | 0,8 | | 0,3 | | 0,6 | | 1,0 | | 1,4 | | 0,0 | | 0,0 | | 0,0 | | 0,0 | | 6,2 | | 4,4 | | 0,3 | | 0,4 | | 0,3 | | 7,5 |
| *Balneolia* | *Balneolaceae* | *Gracilimonas* | 0,9 | | 0,4 | | 0,1 | | 0,2 | | 1,4 | | 1,0 | | 1,3 | | 0,0 | | 0,0 | | 0,0 | | 0,0 | | 3,7 | | 3,2 | | 10,5 | | 3,5 | | 8,0 | | 0,9 | |
|  |  | *Rhodohalobacter* | 1,5 | | 1,3 | | 5,1 | | 9,8 | | 12,9 | | 8,3 | | 20,1 | | 0,0 | | 0,0 | | 0,0 | | 0,0 | | 19,3 | | 22,0 | | 0,6 | | 1,0 | | 0,4 | | 0,4 | |
| *Erysipelotrichia* | *Erysipelotrichaceae* | *Erysipelotrichaceae* UI | 3,5 | | 3,0 | | 0,0 | | 0,0 | | 0,0 | | 0,0 | | 0,0 | | 0,0 | | 0,0 | | 0,0 | | 0,0 | | 0,0 | | 0,0 | | 0,0 | | 0,0 | | 0,0 | | 0,0 | |
| *Alphaproteobacteria* | *Maricaulaceae* | *Oceanicaulis* | 0,0 | | 0,1 | | 5,9 | | 0,0 | | 0,9 | | 1,1 | | 0,8 | | 0,0 | | 0,0 | | 0,0 | | 0,0 | | 0,0 | | 0,0 | | 12,3 | | 2,5 | | 2,1 | | 0,0 | |
|  | *Rhodospirillaceae* | *Marivibrio* | 0,0 | | 0,0 | | 0,0 | | 0,0 | | 0,0 | | 0,0 | | 0,0 | | 99,3 | | 98,0 | | 99,9 | | 30,2 | | 1,4 | | 0,7 | | 0,0 | | 0,0 | | 0,0 | | 0,1 | |
|  | *Parvibaculaceae* | *Tepidicaulis* | 0,0 | | 0,0 | | 0,0 | | 0,0 | | 0,0 | | 0,0 | | 0,0 | | 0,0 | | 0,0 | | 0,0 | | 0,0 | | 1,5 | | 0,1 | | 0,0 | | 0,0 | | 0,0 | | 0,0 | |
|  | *Salinarimonadaceae* | *Saliniramus* | 0,0 | | 0,0 | | 2,9 | | 1,2 | | 2,0 | | 4,1 | | 3,1 | | 0,0 | | 0,0 | | 0,0 | | 0,0 | | 0,0 | | 0,0 | | 0,8 | | 1,6 | | 0,7 | | 0,0 | |
|  | *Roseobacteraceae* | *Roseovarius* | 3,3 | | 5,3 | | 16,5 | | 9,0 | | 18,3 | | 20,0 | | 14,8 | | 0,0 | | 0,2 | | 0,0 | | 0,0 | | 6,2 | | 0,4 | | 6,2 | | 9,4 | | 10,8 | | 1,4 | |
|  |  | *Shimia* | 0,8 | | 1,1 | | 0,0 | | 0,0 | | 0,0 | | 0,0 | | 0,0 | | 0,0 | | 0,0 | | 0,0 | | 0,0 | | 0,0 | | 0,0 | | 0,0 | | 0,0 | | 0,0 | | 0,0 | |
|  |  | *Salibaculum* | 0,8 | | 1,1 | | 0,0 | | 0,0 | | 0,0 | | 0,0 | | 0,0 | | 0,0 | | 0,0 | | 0,0 | | 0,0 | | 0,0 | | 0,0 | | 0,0 | | 0,0 | | 0,0 | | 0,0 | |
|  | *Rhodovibrionaceae* | *Rhodovibrionaceae* UI | 0,0 | | 0,0 | | 20,3 | | 4,4 | | 7,5 | | 8,9 | | 10,3 | | 0,0 | | 0,0 | | 0,0 | | 0,0 | | 15,3 | | 10,3 | | 0,0 | | 0,0 | | 0,0 | | 2,6 | |
| *Betaproteobacteria* | *Alcaligenaceae* | *Bordetella* | 28,0 | | 27,0 | | 0,0 | | 0,0 | | 0,0 | | 0,0 | | 0,0 | | 0,0 | | 0,0 | | 0,0 | | 0,0 | | 0,0 | | 0,0 | | 0,0 | | 0,0 | | 0,0 | | 0,0 | |
| *Gammaproteobacteria* | *Marinobacteraceae* | *Marinobacter* | 0,3 | | 0,3 | | 33,1 | | 28,9 | | 19,0 | | 14,8 | | 6,8 | | 0,4 | | 0,8 | | 0,1 | | 0,0 | | 29,8 | | 10,9 | | 5,7 | | 6,7 | | 10,9 | | 65,4 | |
|  | *Ectothiorhodospiraceae* | *Spiribacter* | 7,5 | | 7,4 | | 13,3 | | 4,5 | | 6,0 | | 8,4 | | 6,1 | | 0,0 | | 0,2 | | 0,0 | | 0,0 | | 2,2 | | 6,9 | | 29,3 | | 40,6 | | 24,3 | | 9,9 | |
|  | *Wenzhouxiangellaceae* | *Wenzhouxiangella* | 2,1 | | 1,9 | | 0,1 | | 0,5 | | 0,4 | | 0,9 | | 0,4 | | 0,0 | | 0,0 | | 0,0 | | 0,0 | | 0,0 | | 0,0 | | 0,0 | | 0,0 | | 0,0 | | 0,0 | |
|  | *Alcanivoracaceae* | *Alloalcanivorax* | 0,0 | | 0,0 | | 0,0 | | 0,0 | | 0,1 | | 0,0 | | 0,0 | | 0,2 | | 0,0 | | 0,0 | | 69,7 | | 0,0 | | 0,3 | | 0,0 | | 0,0 | | 0,0 | | 11,8 | |
|  | *Halomonadaceae* | *Halomonas* | 0,4 | | 0,9 | | 0,0 | | 0,0 | | 0,3 | | 0,6 | | 0,1 | | 0,0 | | 0,0 | | 0,0 | | 0,0 | | 0,0 | | 0,1 | | 26,1 | | 25,6 | | 21,5 | | 0,0 | |
|  | *Piscirickettsiaceae* | *Methylophaga* | 0,1 | | 0,1 | | 0,0 | | 0,0 | | 1,7 | | 0,5 | | 0,2 | | 0,0 | | 0,0 | | 0,0 | | 0,0 | | 11,6 | | 39,2 | | 2,2 | | 3,9 | | 6,2 | | 0,0 | |
| *Spirochaetia* | *Spirochaetaceae* | *Spirochaetaceae* UI | 0,0 | | 0,0 | | 1,6 | | 0,7 | | 0,3 | | 0,3 | | 0,1 | | 0,0 | | 0,0 | | 0,0 | | 0,0 | | 0,0 | | 0,0 | | 0,9 | | 0,1 | | 0,1 | | 0,0 | |
| *Bacteria* UI | | | | 0,1 | | 0,1 | | 0,1 | | 0,1 | | 0,0 | | 0,0 | | 0,0 | | 0,0 | | 0,0 | | 0,0 | | 0,0 | | 2,4 | | 0,9 | | 4,6 | | 4,2 | | 1,5 | | 0,0 |
| *Opitutia* | *Coraliomargaritaceae* | *Coraliomargarita* | 0,1 | | 0,1 | | 0,0 | | 35,3 | | 17,8 | | 15,0 | | 15,4 | | 0,0 | | 0,0 | | 0,0 | | 0,0 | | 0,0 | | 0,0 | | 0,0 | | 0,0 | | 0,0 | | 0,0 | |
| others | | | | 7,5 | | 7,5 | | 0,3 | | 0,4 | | 0,5 | | 1,2 | | 0,7 | | 0,1 | | 0,7 | | 0,0 | | 0,0 | | 0,4 | | 0,5 | | 0,3 | | 0,2 | | 0,2 | | 0,0 |

**Table S3.** Numbers of raw reads, high-quality reads, and taxonomic richness in DNA libraries from the prokaryotic community of the ephemeral pond near the Solyanka River and the prokaryotic assemblages associated with the derived monoalgal cultures

| DNA library number | Water sample or microalga species | Total reads | High-quality reads | Reads analysed | Number of OTUs | Number of genera | Number of classes | Number of phyla | % of reads, aligned to those in the water sample |
| --- | --- | --- | --- | --- | --- | --- | --- | --- | --- |
| 40z_214 | ephemeral pond near Solyanka River | 40,569 | 20,012 | 23,842 | 196 | 97 | 26 | 15 | 100.0 |
| Sol_17z_18 | *Asteromonas gracilis* | 44,351 | 20,924 | 24,716 | 74 | 43 | 9 | 5 | 90.6 |
| Sol_17z_19 | *Asteromonas gracilis* | 37,852 | 17,476 | 22,351 | 77 | 43 | 9 | 4 | 91.1 |
| Sol_17z_27 | *Asteromonas gracilis* | 46,614 | 24,509 | 24,008 | 71 | 42 | 10 | 5 | 77.4 |
| Sol_17z_28 | *Asteromonas gracilis* | 51,918 | 27,381 | 26,671 | 77 | 45 | 10 | 5 | 71.3 |
| Sol_17z_20 | *Tetraselmis indica* | 46,265 | 20,708 | 30,121 | 61 | 40 | 8 | 4 | 91.7 |
| Sol_17z_21 | *Tetraselmis indica* | 35,107 | 18,031 | 22,562 | 47 | 26 | 4 | 3 | 86.3 |
| Sol_17z_22 | *Tetraselmis indica* | 40,019 | 20,843 | 32,802 | 30 | 21 | 4 | 3 | 88.0 |
| Sol_17z_29 | *Tetraselmis indica* | 47,606 | 25,975 | 38,185 | 66 | 37 | 8 | 6 | 97.2 |
| Sol_17z_23 | *Dunaliella sp.* | 45,721 | 22,411 | 34,723 | 38 | 21 | 7 | 5 | 99.6 |
| Sol_17z_24 | *Dunaliella sp.* | 44,176 | 21,902 | 30,538 | 36 | 19 | 4 | 3 | 99.8 |
| Sol_17z_25 | *Dunaliella sp.* | 41,721 | 21,081 | 29,159 | 33 | 18 | 5 | 3 | 99.9 |
| Sol_17z_26 | *Dunaliella sp.* | 37,886 | 19,254 | 24,623 | 30 | 14 | 3 | 2 | 99.9 |
| Sol_17z_17 | Control | 28,898 | 13,270 | 18,894 | 21 | 12 | 5 | 3 | 99.9 |
| Sol_17z_31 | Control | 49,543 | 25,916 | 39,368 | 37 | 19 | 6 | 4 | 99.9 |

**Table S4.** Numbers of raw reads, high-quality reads, and taxonomic richness in DNA libraries from the prokaryotic community of the Malaya Samoroda River and the prokaryotic assemblages associated with the derived monoalgal cultures

| DNA library number | Water sample or microalga species | Total reads | High-quality reads | Reads analysed | Number of OTUs | Number of genera | Number of classes | Number of phyla | % of reads, aligned to those in the water samples |
| --- | --- | --- | --- | --- | --- | --- | --- | --- | --- |
| 40z_216 | Malaya Samoroda River 1 | 31,441 | 13,454 | 14,619 | 134 | 75 | 25 | 14 | 100.0 |
| 40z_215 | Malaya Samoroda River 2 | 37,878 | 17,756 | 11,075 | 117 | 70 | 23 | 13 | 100.0 |
| MS_17z _1 | *Navicula sp.1* | 47,609 | 16,722 | 30,335 | 29 | 15 | 6 | 5 | 76.0 |
| MS_17z _3 | *Navicula sp.1* | 32,671 | 15,118 | 12,386 | 33 | 16 | 8 | 6 | 93.5 |
| MS_17z _4 | *Navicula sp.2* | 10,432 | 4,020 | 5,245 | 34 | 20 | 7 | 5 | 89.6 |
| MS_17z _5 | *Navicula sp.2* | 48,721 | 20,225 | 35,645 | 43 | 24 | 8 | 5 | 86.5 |
| MS_17z _6 | *Navicula sp.2* | 47,462 | 18,313 | 27,136 | 46 | 24 | 8 | 6 | 85.7 |
| MS_17z _7 | *Tetraselmis indica* | 32,917 | 11,048 | 2,329 | 4 | 4 | 2 | 1 | 99.7 |
| MS_17z _8 | *Tetraselmis indica* | 46,186 | 21,147 | 1,658 | 5 | 5 | 2 | 1 | 99.0 |
| MS_17z _16 | *Tetraselmis indica* | 57,492 | 17,047 | 5,069 | 2 | 2 | 2 | 1 | 100.0 |
| MS_17z _119 | *Tetraselmis indica* | 55,734 | 28,238 | 17,437 | 3 | 3 | 2 | 1 | 30.2 |
| MS_17z _120 | *Picochlorum sp.* | 80,277 | 36,431 | 28,974 | 38 | 16 | 5 | 4 | 81.7 |
| MS_17z _121 | *Picochlorum sp.* | 61,199 | 23,367 | 24,197 | 38 | 18 | 8 | 6 | 85.7 |
| MS_17z _122 | *Picochlorum sp.* | 58,082 | 25,262 | 19,258 | 40 | 18 | 8 | 6 | 98.5 |
| MS_17z _123 | *Picochlorum sp.* | 68,424 | 33,126 | 29,354 | 41 | 15 | 8 | 6 | 98.0 |
| MS_17z _11 | *Picochlorum sp.* | 35,921 | 16,849 | 22,388 | 27 | 15 | 5 | 4 | 99.3 |
| MS_17z _13 | Control | 34,635 | 14,641 | 29,981 | 23 | 11 | 4 | 3 | 84.8 |
